# Application of an ensemble CatBoost model over complex dataset for vehicle classification

**DOI:** 10.1371/journal.pone.0304619

**Published:** 2024-06-12

**Authors:** Pemila M., Pongiannan R. K., Narayanamoorthi R., Kareem M. AboRas, Amr Youssef

**Affiliations:** 1 Department of Electrical and Electronics Engineering, SRM Institute of Science and Technology, Kattankulathur, Chengalpattu, India; 2 Department of Computing Technologies, SRM Institute of Science and Technology, Kattankulathur, Chengalpattu, India; 3 Department of Electrical Power and Machines, Faculty of Engineering, Alexandria University, Alexandria, Egypt; 4 Electrical Engineering Department, University of Business and Technology, Ar Rawdah, Jeddah, Saudi Arabia; University of Manitoba, CANADA

## Abstract

The classification of vehicles presents notable challenges within the domain of image processing. Traditional models suffer from inefficiency, prolonged training times for datasets, intricate feature extraction, and variable assignment complexities for classification. Conventional methods applied to categorize vehicles from extensive datasets often lead to errors, misclassifications, and unproductive outcomes. Consequently, leveraging machine learning techniques emerges as a promising solution to tackle these challenges. This study adopts a machine learning approach to alleviate image misclassifications and manage large quantities of vehicle images effectively. Specifically, a contrast enhancement technique is employed in the pre-processing stage to highlight pixel values in vehicle images. In the feature segmentation stage, Mask-R-CNN is utilized to categorize pixels into predefined classes. VGG16 is then employed to extract features from vehicle images, while an autoencoder aids in selecting features by learning non-linear input features and compressing representation features. Finally, the CatBoost (CB) algorithm is implemented for vehicle classification (VC) in diverse critical environments, such as inclement weather, twilight, and instances of vehicle blockage. Extensive experiments are conducted using different large-scale datasets with various machine learning platforms. The findings indicate that CB (presumably a specific method or algorithm) attains the highest level of performance on the large-scale dataset named UFPR-ALPR, with an accuracy rate of 98.89%.

## 1. Introduction

In contemporary urban environments, surveillance cameras have become ubiquitous. The primary purpose of deploying these surveillance systems revolves around two key objectives: real-time monitoring and event retrieval [1, 2]. This paper exclusively focuses on the latter, emphasizing the importance of event retrieval for law enforcement agencies. For instance, it aids police officers in searching for specific vehicles [3, 4]. To accomplish this task effectively, officers require detailed information about the vehicle’s characteristics, including its color and type, which serve as essential clues for vehicle identification [5, 6]. Unfortunately, officers often invest a substantial amount of time manually monitoring recorded videos. Typically, the time spent on searching exceeds the duration of the video itself, necessitating multiple repetitive search attempts. Furthermore, fatigue can set in after prolonged searching, misclassification, potentially leading to errors in the process [7, 8]. The existing system suffers from inaccuracies and errors due to manual operations conducted by individuals. These inaccuracies in the system result in financial losses for individuals employed by the supplier. This paper sparks innovative solutions for addressing existing problems [9]. The paper aims to develop an efficient real-time VC system by leveraging ten predominant large-scale vehicle datasets [10]. To facilitate VC based on distinctive features, the paper employs the CB algorithm, which provides a gradient-boosting framework [11, 12]. This algorithm introduces a unique approach for handling categorical features through a permutation-driven method, deviating from conventional algorithms. The computer vision methodology for VC predominantly focuses on preprocessing, feature selection, feature extraction, and the classification process. Image processing is used for recognizing patterns whereas machine learning used to train system to identify pattern changes [13]. Thus, image processing using machine techniques plays a vital role in the modern era for VC. Initial image data is sourced from various large-scale datasets, including Stanford car, vehicle rear, MVVTR, Indian Vehicle, TRANSCOS, Thai vehicle classification, 2023 Car Model, UFPR-ALPR, Vehicle x and CompCar datasets [14, 15]. After the acquisition of the vehicle dataset, the extraction and curation of vehicle features are performed. These selected features are instrumental in enhancing the training process for the classifier, resulting in more precise vehicle categorization [16, 17]. The features encompass both global and local descriptors. The initial stage in the VC workflow involves preprocessing [18, 19]. Directly employing raw data or images for classification is unfeasible due to the potential for inaccuracies arising from image noise, geometric distortions, variations in image size, and color inconsistencies [20, 21]. Thus, preprocessing techniques are employed where the noise reduction, and pixel quality improve. The background is eliminated, and specific vehicle location are identified [22]. The result of the pre- processing technique will be fed as input to the feature segmentation. Under feature segmentation, the vehicle images are masked so that the pixel are classified to pre-defined classes [23, 24]. After feature segmentation, the feature extraction process is carried out. In this feature extraction method, the large training data are handled so that the trained data can be fed into next stage, namely feature selection. Followed by feature extraction, feature selection process is performed. In this feature selection process, accurate features are defined. Once accurate features are defined, the vehicles images are classified from others based on classes, types and model using classification algorithm in the classifier model. The classifier algorithm plays a vital role in classification of vehicles. Considering mathematical and statistical parameters, the vehicle images are classified from the large-scale dataset [25, 26]. There are many classifiers’ algorithms support vector machine (SVM), decision making, Naïve Bayes (NB), K-nearest neighbor (KNN), k-means clustering, logistic regression (LR), linear discriminant analysis, random forest (RF), cluster analysis and quadratic equation [27, 28]. Out of these classifier algorithms, decision trees in machine learning furnish a strong method for providing decision since it knocks out the problem of possible outcome. Overfitting, error bias and variance error are drawbacks of decision trees [29]. These are overcome by the gradient boosting algorithm, where classification and regression can be found. There are four types of boosting algorithm in machine learning namely Gradient Boosting, extreme gradient boosting (XGBoost), light gradient boosting (LGB) and CB machine [30, 31]. Out of these, CatBoost has notable feature of more accurate than any other mode of classifier, train faster in large scale dataset, handling the missing values, supporting categorical features, training performed on multiple GPUs, good performance in assumption parameters, fast prediction and hold up with classification and regression problem [32]. VC from large scale dataset is great challenging in computer task. In this paper, different large-scale datasets, namely Stanford car, Vehicle rear, MVVTR, Indian Vehicle, TRANSCOS, Thai VC, 2023 Car Model, UFPR-ALPR, Vehicle x and CompCar datasets are utilized for VC [33, 34].

The major primary contributions of this paper can be succinctly outlined as follows:

- Contrast enhancement technique is used for pre-processing technique used to differentiate image features which outline the pixel values and improve the quality images.
- Mask–R-CNN for feature segmentation which segment mask of the vehicle and also classify the pixel into pre-defined categories.
- Random forest technique is used for feature selection method for improve purity of node.
- CatBoost algorithm for classification technique to classify the vehicle from large scale dataset.

The subsequent sections of this paper are structured as follows: Section II delves into the discussion of related works and outlines the problem description. Section III elucidates the research methodology pertaining to the proposed approach. A detailed presentation of the VC methods employing the CatBoost algorithm is expounded in Section IV. Comprehensive experiments and in-depth analysis are conducted in Section V. Finally, Section VI provides a summary of the research endeavors.

## 2. Related works

This section highlights the most recent work related to VC over large scale dataset using classifier technique has been reviewed. Due to poor installation of camera, bad weather conditions and occlusion of vehicle lead to inaccuracies in classification of vehicle. VC majorly concern on feature extraction and classification using classifier. The classifiers are trained with extracted features and model is used to classify the vehicles from large scale dataset [35]. Since vehicles are classified using raw data and video frame, there are the chances of leakage of vehicle owner confidential, hence the size of bus, car and motorcycles using seismic waves are considered. It is compared with LR, SVM and NB. In this scenario, NB gain F1 score upto 97%. But traffic flow, speed, direction and driver safety are not considered. No bicycles are considered, and only single dataset are taken [36, 37]. In case of vehicle re-identification, vehicle databases are gained by integration of global and local features, channel attention module is used for feature extraction, using weighted feature the noise and background are eliminated. The drawback in this paper is that only three datasets are considered for vehicle re-identification. Light motor vehicles are not considered [38, 39]. Using SVM and OCR the vehicles are classified with the accuracy of 98.3% but single dataset is taken [40, 41]. Using sensor (IR and ultrasonic) the vehicle is detected as sedan, pick up, SUV and two-wheeler with the accuracy of 99%. The accuracy of classification of vehicle based on classifier is not mentioned with more precisely [42]. 100% accuracy VCs are performed using SSD method, but the camera is kept on top of another vehicle with shorter and single dataset are considered. DAWN, CDNet 2014, LISA 2010 dataset are considered for vehicle detection using improved version of YOLO but it gain the accuracy of 95.67% [43]. Edge-empowered Cooperative Multi-Camera sensing system is proposed for vehicle tracking over two vehicle datasets with the maximum accuracy of 92.43%. But the limitation are, it does not work on severe climatic conditions [44, 45]. Using classifier like Naïve Bayes, vehicle faults [46] are identified with the features of temperature, noise and vibration of the vehicles even parking management system [47] are performed. Similarly using KNN classifier, from 4 type of scenario, vehicle classified with the help of forward scattering radars and gain accuracy of 99% [48]. Only car signatures are taken consideration [49]. Whereas for the fast classification of identifying vehicle from road, Light GBM, KNN and SVM are used to classify the vehicles. Under these trails, Light GBM classify the vehicle with a faster rate of 0.015 sec. [50] with the decision trees, the vehicle are classified with the accuracy of 99.38% [51]. Hence to boost the accuracy in the vehicle detection, XGBoost classifier is used. But it gains accuracy of 97.07% having single dataset. XGBoost used to classify two-wheeler including e-bikes from two large scale dataset with the accuracy of 99% [52]. For prediction analysis, Random Forests and Deep Learning Neural Network are used, which gain maximum accuracy of 96.6% [53]. Using WIFI, the vehicles are classified which gain accuracy upto 100%, it has limitation of extracting peak value of the signal. If the signal is weak, there would be a misclassification rate [54, 55]. Concerning the features of vehicles like color and shapes from vehicle dataset, the vehicles are classified using CatBoost algorithm. Light motor vehicles over two datasets are considered for VC. In some cases, hybrid models are used for detection purposes. In [56] this author proposes, hybrid model CNN and CatBoost utilizes to classify test samples to make prediction with the accuracy of 96.15%. A hardware accelerator named FPGA is used for faster processing speed with low power for VC [57], lane detection, traffic signal and obstacle detection [58]. In this more features are considered for classification of the vehicles. In Table 1, the performances of the classifier over classification are summarized.

**Table 1:** Summary of the classifier performances over VC.

S.No	Authors	Dataset /Number	Features considered	Classes/ model/types	Accuracy	Problem identified	Solution
1	(8)	2	4	3	90%	High cost, limited range detection and hard to providing real time traffic information.	GMM, Optical flow and ACF is utilized
2	(9)	1	930 samples	4	97%	Camera has big issues of poor weather.	LR, SVM and Naïve bayes used
3	(10)	3	2	4	98.11%	Poor learning capacity of model	Fusion of features
4	(11)	1	4	6	98.3%	Poor local optima and long frame	SVM classifier used for maximum likehood of generalised model
5	(12)	1	103833 images	7	97.84%	More training time	CNN layers is reduced
6	(13)	1	3	4	91.75%	More computation time	SSD is used for reduction of Frame size
7	(14)	3	47 fps	9	95.75%	Detection of bounding box consume more time	Improved yolo to detct vehicle through regression
8	(15)	2	4	4	93.72%	Low accuracy	Raw video into edge node
9	(16)	1	500 features	6	90.67%	Detection is less accuracy	NB to detect with more precise
10	(17)	1	4	3	Around 96%	Larger stimulation time	NB provide better accuracy
11	(18)	1	5	6	99%	Noise in pre-processing technique	KNN algorithm for classification accuracy
12	(19)	4	3	3	Around 95%	Faster detection	SVM classifier for earlier detection

Thus, from the literature survey, it concludes that there are limitations like range extenders and insufficient mass-produced models in traditional dataset namely Cars-Conventional engine and EVs. Even, Bangladeshi vehicle dataset face significant challenges in classifying vehicle images when deployed in model like VGG19, ResNet-152. The existing vehicle dataset has issues on intra-class, scalability, and angle variation. This leads to poor accuracy in classifying the vehicles from large scale vehicle dataset. Similarly, similar sizes and color of the vehicles are hard to distinguish the vehicle category. Noise and unwanted features also occur in the feature extraction process. Therefore, the proposed technique is implemented to overcome all these challenges by employing ensemble algorithms, namely CatBoost algorithms. This classifier is used to categorize vehicle images into one more class from large scale dataset. Reduction of high dimensionality features, minimum model training time, simple model which gain the VC accuracy. This approach results in a remarkable accuracy rate of 98.89% in VC, particularly using CatBoost algorithm, on a large-scale dataset.

## 3. Material and methods

**Fig 1:**
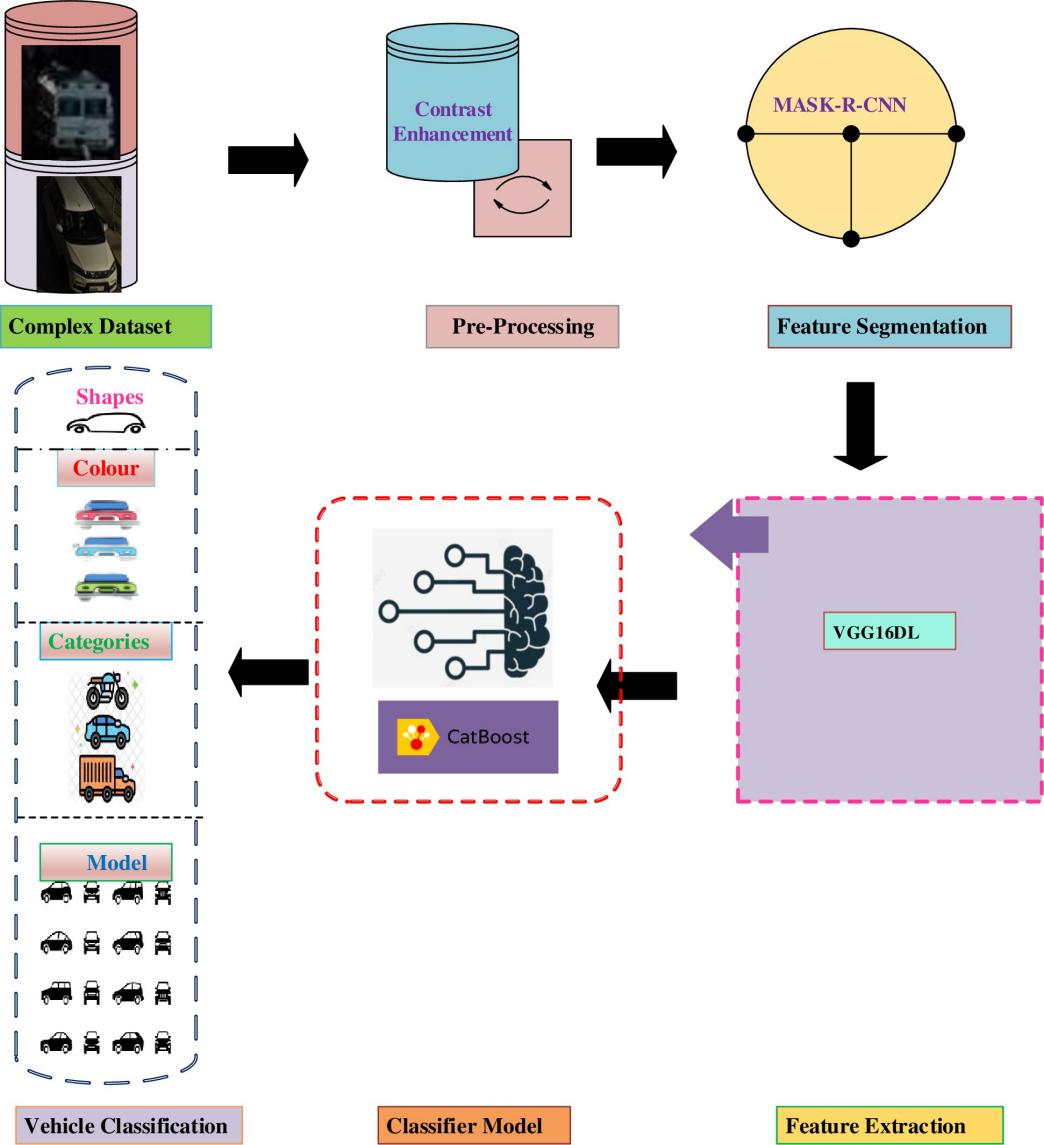
An outline of the envisaged framework for vehicle classification.

Vehicle dataset is the collection of vehicle images of different make and model using various modes like digital single lens reflex, camcorder, and mobile phones irrespective of various time, location and weather conditions. From large scale vehicle dataset, the vehicle classes and label’s information are extracted with advanced technologies to classify the vehicle as size, shape, color, make and model. Various vehicle factors and parameters can be predicted with the development of machine learning techniques. To achieve the precise classification in visual content from a large-scale dataset, an ensemble algorithm is utilized. This encompasses various processes such as preprocessing, feature extraction, segmentation, selection, and the use of a classifier. Fig 1 illustrates the block diagram of the proposed VC using CB.

In Fig 1 An outline of the envisaged framework for vehicle classification in adverse weather conditions is provided. Vehicle features are derived through the utilization of pre-trained VGG16, denoted by the pink dotted line. The subsequent vehicle categorization is executed through the CB algorithm, represented by the red dashed line. The blue dashed line illustrates the process of classifying vehicles into various forms.

### 3.1. Acquisition of vehicle datasets

**Fig 2:**
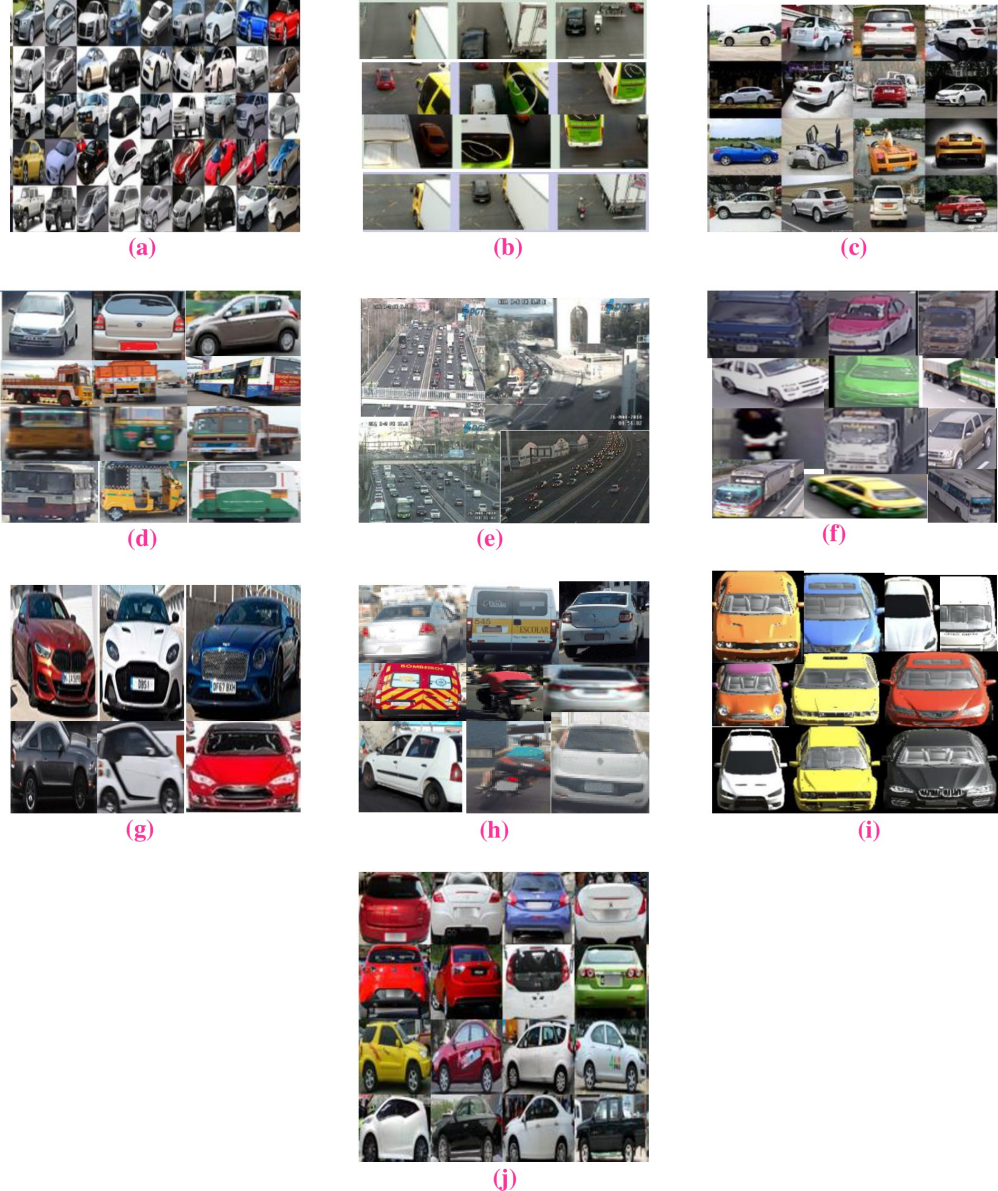
Sample images from various vehicle datasets. (A) Stanford car dataset (B) Vehicle–Rear dataset (C) MVVTR dataset (D) Indian Vehicle dataset (E) TRANCOS dataset (F) Thai Vehicle Classification Dataset (G) 2023 Car Model dataset (H) UFPR-ALPR dataset (I) VehicleX dataset (J) CompCars dataset.

Vehicle datasets are governed with vehicle data collection obtained from observation, calibration, and analysis in form of data. The information is in the form of numeric, figures, label, or basic description. The vehicle images are fetched from the site of Google open images and kaggle competition. The list of vehicle datasets is mentioned as follows for proposed VC. In Fig 2, portray the collection of various large scale vehicle dataset for VC.

#### 3.1.1 Stanford car dataset

The dataset contains nearly 16K images of different categories of car. Make, model and year of the vehicle are categorized. It also contains 3D orientations for multi view object class identification. (https://www.kaggle.com/datasets/jessicali9530/stanford-cars-dataset).

#### 3.1.2 Vehicle–Rear dataset

Vehicle—rear dataset is used for vehicle identification having HD videos with more precise information of make, model, color and year of the vehicles. It is a novel dataset containing 3k vehicles and it further used to for identification and position of concern vehicle license plates. (https://paperswithcode.com/dataset/vehicle-rear).

#### 3.1.3 MVVTR dataset

Multi view VTR contains 7K real vehicles images with different types. Each type has 1K images. Images are taken from different angles hiding the license plates for security purposes. Images are collected from internet search engines and vehicles images are labelled. (https://ieeexplore.ieee.org/stamp/stamp.jsp?tp=&arnumber=8004504).

#### 3.1.4 Indian vehicle dataset

The Indian vehicle consist of images containing vehicle which is used for VC and identifying the objects. The images contain various types of vehicles. The images have been taken under different climatic situations. It has different illumination, distances and viewpoints of vehicle images and also used for image recognition and object detection for autonomous driving driver. (https://www.kaggle.com/datasets/dataclusterlabs/indian-vehicle-dataset).

#### 3.1.5 TRANCOS dataset

Traffic and congestion (TRANCOS) dataset handles overlapping vehicle in traffic situations used for vehicle counting. It consists of 1244 images, with 46796 vehicles annotated. The images are taken from the CCTV provided by Spain government. (https://gram.web.uah.es/data/datasets/trancos/index.html).

#### 3.1.6 Thai vehicle classification dataset

The dataset prepared from road maintenance unit under department of rural road of Thailand. It contains 6.3TB of videos from 23 cameras for 3 days in the year 2020. The number samples or class is car, bus, taxi, bike, pick up, truck and trailer. The total number of samples is 29474. (https://www.linkedin.com/pulse/thai-vehicle-classification-dataset-bipul-neupane/).

#### 3.1.7 2023 car model dataset

The 2023 car model dataset includes a comprehensive collection of information about cars. The diesel includes horsepower rate, torque value, types of vehicle transmission, and numbers of doors, price, model and body of the vehicles. (https://www.kaggle.com/datasets/anoopjohny/2023-cars-dataset).

#### 3.1.8 UFPR-ALPR dataset

The UFPR-ALPR has 4K vehicles images with different angles containing 30K characters from existing conditions. The vehicle as well as the camera are on mobility. The specification of vehicle images is 1920x1080 pixels. GoPro Hero4Silver, Huawei P9 lite and iPhone 7 Plus cameras are utilized. (https://web.inf.ufpr.br/vri/databases/ufpr-alpr/).

#### 3.1.9 VehicleX dataset

VehicleX is a complex vehicle dataset which is scalable. It contains 1362 vehicles images of various 3D models with fully editable attributes. (https://paperswithcode.com/dataset/vehiclex).

#### 3.1.10 CompCars dataset

CompCars dataset contains both web and surveillance images. The cross-modality analyses of car can perform. The dataset contains nearly car images of 136727 and 27K images of its parts. The hierarchy of attributes and viewpoints of the car are their unique features. (https://mmlab.ie.cuhk.edu.hk/datasets/comp_cars/).

In Fig 2 observe that there is great diversity in term of climatic condition, poor quality of images, diverse in vehicle type, shape, color and models, occlusion, reflection and resolution.

### 3.2 Pre-processing

Once the dataset is collected it is sent to the preprocessing process. Since raw images fed into classifier model led to provide poor result of image classification. Thus, for noise removal, the preprocessed techniques are enhanced to boost the vehicle image features. An efficient method, Contrast enhancement techniques is used for differentiating various vehicle image features from input images. This method will increase the image contrast of digital image by outlining the pixel values to a comprehensive. It improves the visibility of details and features in vehicle images. This technique is image magnification in which quality of an image are improved by expanding image intensity values. In this, need to stretch the minimum and maximum values of intensity to maximum extent to avoid poor quality image preprocessing. The contrast stretching formula is given by,

$$C=(v-v_{min})+\left(\frac{y_{max}-y_{min}}{v_{max}-v_{min}}\right)+y_{min}$$ (1)

**Fig 3:**
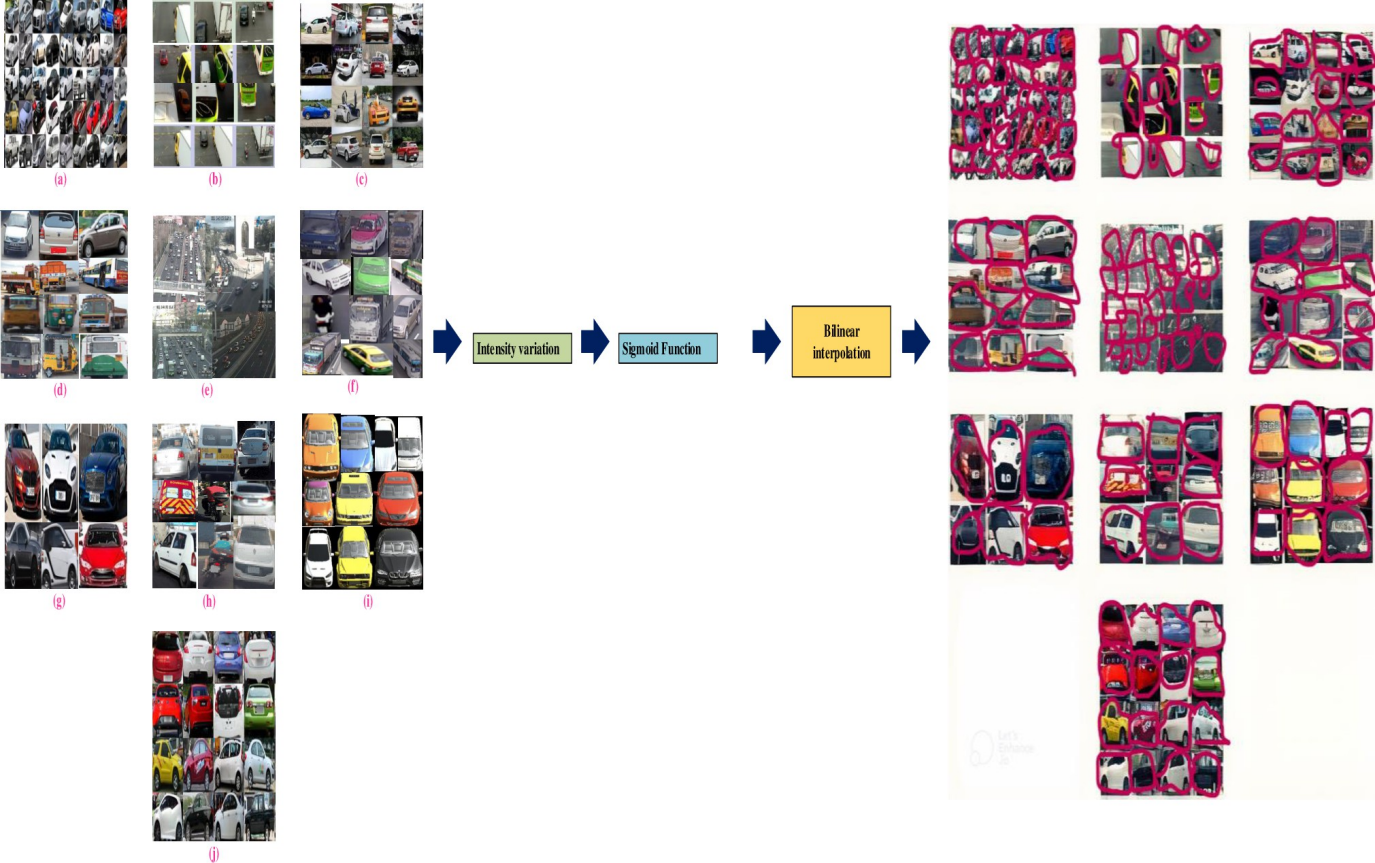
The intensity variation map.

The Fig 3 indicates the original vehicle dataset, the intensity variation map is computed. From the computation output sigmoid map function are evaluated for individual contextual region. The intensity transforms into real vehicle image by linear interpolation. The vehicle images undergo enhancement through the application of contrast enhancement techniques, denoted by the highlighting in pink.

In Eq (1), the existing pixel intensity value is given by *v*, *v*_*min*_ and *v*_*max*_ denotes the minimum and maximum intensity values lies in the entire image respectively. The output result is shown in Fig 3 using proposed pre-processing technique namely contrast enhancement method.

### 3.3 Feature segmentation

Feature segmentation is classifying the pixel into classes. In this paper Mask R-CNN, is used for vehicle feature segmentation. Image segmentation generates a pixel-wise mask for individual vehicles provided in the image. image. Class label, bonding box coordinates for each vehicle for masking. Initially, features are evoked from the fetched image using ResNet architecture. These features are then fed as input to the next layer to obtain the feature map. The mapped features are enforced to the region proposal. These predict the vehicle location in the image. The regions are of various shapes. By adding pooling layer, all regions are converted into similar shape. After region is passed in fully connected network, class label, bounding box is predicted. In further, the Mask R-CNN generate the segment mask. The RoI is performed for quick computation time. The IoU with ground truth box is calculated. IoU is calculated using (2)

IoU=AoI/AoU (2)

**Fig 4:**
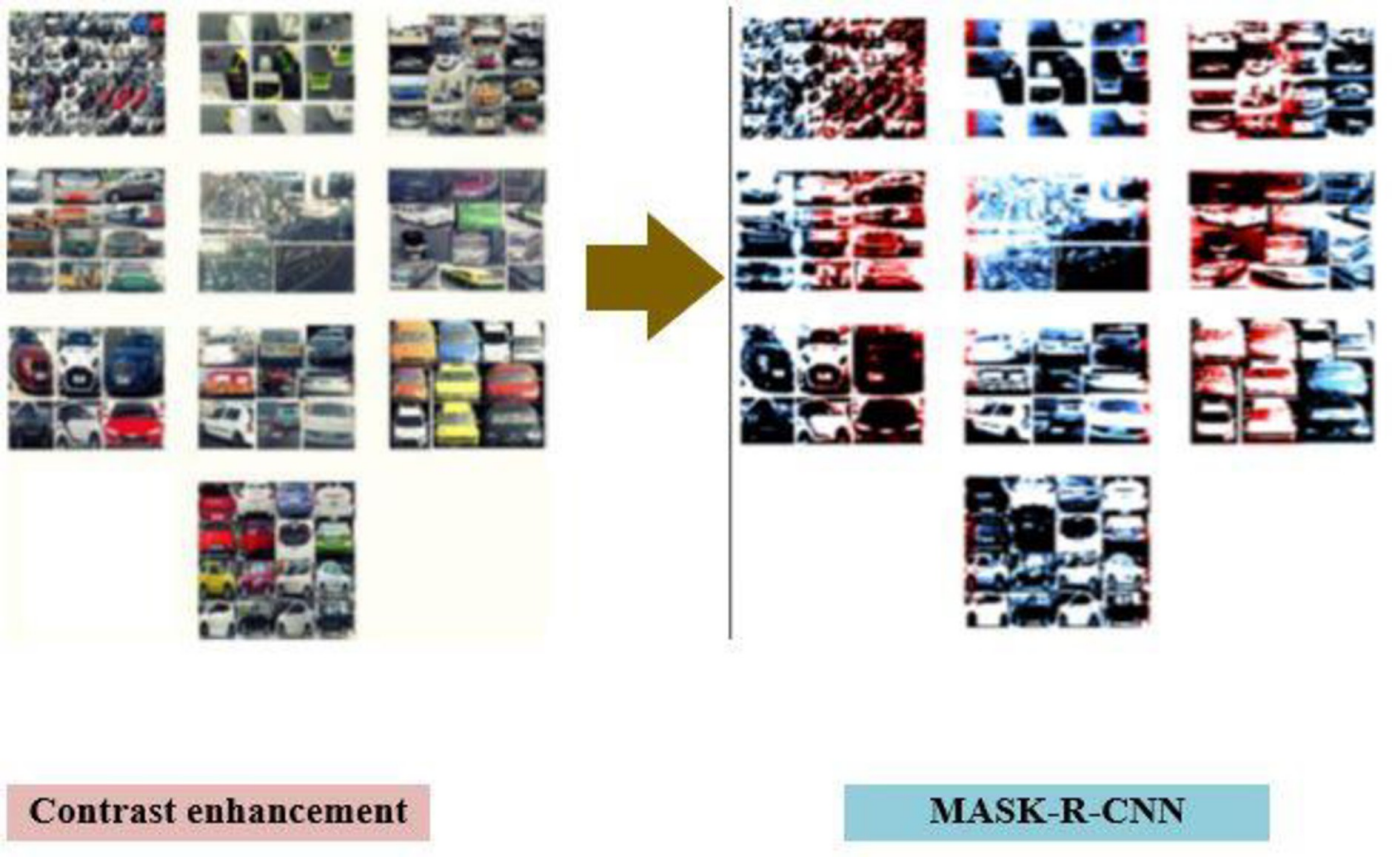
Prediction of each segmentation mask over region of interest.

Where AoI is the area of intersection, AoU is the area of union. The IOU should be more than or equal to 0.5, in that case RoI can neither be considered or not. Presiding, region of interest (RoI) based intersection over union (IoU), the mask is included in the current framework. The return segmentation mask for each region contains vehicle images that scaled up for inference. Thus, vehicle images are predicted with the mask. Thus, in Fig 4 the MASK-R-CNN output is shown.

Fig 4 indicates the prediction of each segmentation mask over region of interest of enhance vehicle images which preserve the spatial information to form a map with fixed size.

### 3.4 Feature extraction

Feature extraction is a dimensionality depletion method. Next to mask prediction of vehicle images, feature extraction process is executed. In these processes, the best feature is choosing from large scale vehicle dataset by preferring and fusing vehicle variables into features which lead to reduce the quantity of data. This will avoid the large computing time. For further process, it is more feasible to acquire accuracy in original form. To identify an exact feature extraction and conversion, several methods are used such as linear discriminant analysis, principal component analysis and kernel principal component analysis. The VGG16 model of feature extraction provides good accuracy from large data. In recent trends, one of the most popular techniques of image feature extraction is VGG16 which perform better than VGG19 and Alexnet deep learning (DL) model in the application of classification tasks. VGG19 requires more weight in network compared to VGG16. Similarly, the large-scale dataset with deeper network layer advocate the processing with lesser filter give rise to ameliorate performance system when compared to Alexnet [59–61].

Thus, in this paper, VGG16 DL model is implemented for feature extraction given proposed work. The VGG16 model is implemented on collection of vehicle datasets. The extracted features are mainly shapes, color, textures, and text. The features of vehicle internal characteristics like number of seat, internal load capacity are deprived. The features which have low level characteristics. The network can be able to learn the feature when the classes are unvaryingly distributed. The unbalanced dataset is categorized with the bias function. The uneven distribution is useful for better performing more classes of vehicle images. The proposed model utilizes diverse layers for feature extraction. The convolution, fully connected and pooling layer are a major part of the architecture. The convolution and fully connected layer consist of 16 layers. 224*224 pixels is the input size of the network with 3*3 filter size. Finally, the activation function can afford probabilities of classes to output layers. The foremost step for feature extraction is providing 224*224-pixel images to the first layer of the VGG16 network. Followed by fetching of images, the images are fed to various convolutional layers. The padding and stride value fixed at one. The architecture maintains entire spatial resolution to match the base images with the activation dimensions. The functions are processed to pooling layer with 2 pixel of stride value with the window size 2x2. The activation size gets reduced by ½.

**Fig 5:**
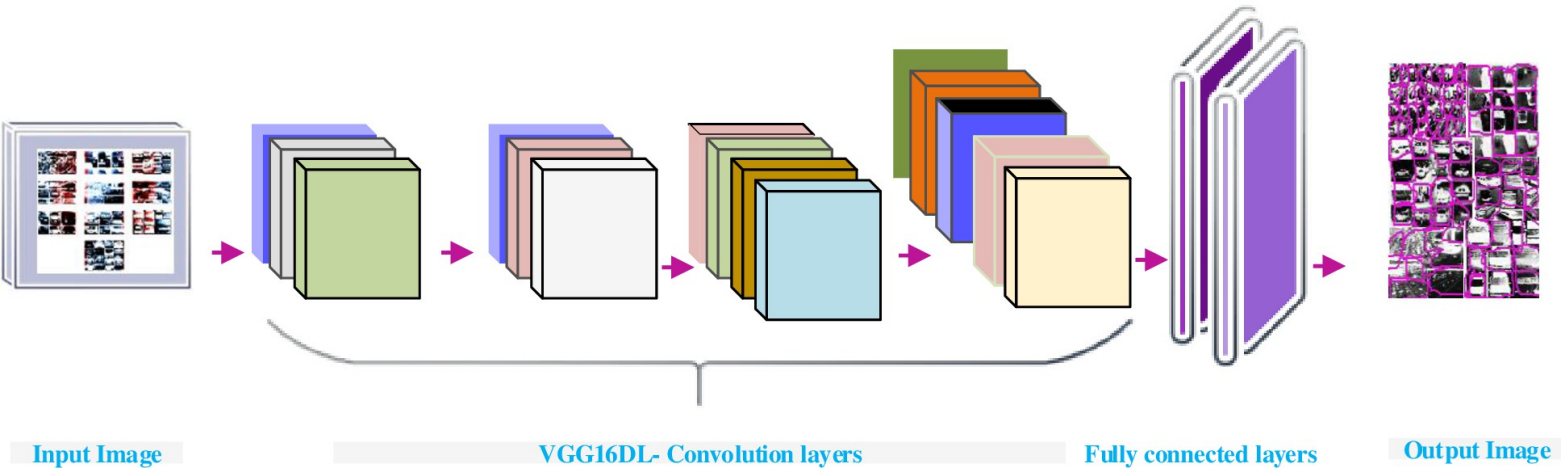
The segmented mask images, the pixel values are scaled up to model based on array processing.

Additionally, in the second step output is again fed into convolutional layers for stack purpose. The output is 56*56*128 pixels. Thus, the last layer provide result upto 1000 classes. The performance is compared with the GhostNet, ResNet10, ResNet50 and VGG16 is explained in section 4. The vehicle extraction using VGG16 is demonstrated in Fig 5.

### 3.5 Feature selection

Feature selection is the process of nominating the most essential features in the image dataset that provide optimal model performance in machine learning. This technique is used to avoid confusion between models, boost performance of model and interpretability. There are various methods of feature selection such as filter, wrapper and embedded. But recently auto encoders have remarkable approach on compression and reconstruction of images. The filter method lack deeper understanding of data, wrapper method make model intensive computation and embedded method feel hard to hold high dimensional data. To overcome the above stated issues, the auto encoder is implemented.

Initially, the dataset consists of vehicle measurement like dimensions, color, types and speed of various vehicles. Each type of vehicle is labelled properly which is suitable for supervised learning. Loading and pre-processing data to make ready to feed into model. Vehicle dataset and standardize the dataset to ensure that features have a mean 0 and standard deviation of 1. This standardization helps us to merge faster during training and features must be on an identical scale [62–64]. Followed by standardization, the data is separated into training and testing set. An auto encoder is constructed, where the neural network learns to compress and reconstruct the input data. The architecture layer is defined as input, encoding and decoding layer. In the input layer, the number of features in dataset is matched, whereas in encoding layer, the data dimension is reduced. Finally, in decoding layer reconstruct the input from encoded representation. After reconstruction model, auto encoder feed data into model and train model on vehicle dataset. The network compressed and learned the features from dataset. This technique requires dependent and independent variable, number of epochs, batch size and parameters. The models start for training for number epoch. After training auto encoder, the important features are extracted. Finally, selected features are integrated with predictive model using machine learning algorithm to calculate the performance of feature selection using autoencoders. The autoencoders model separate encoder and decoder part [65, 66]. The encoder is used as forefront of any model to classify images. The vehicle images are fed to an encoder for compression of information vector, and this will be input to the classification model. In the case of deep learning model, the deep features were minimized to 1000 to 100 by using less redundancy with highest related algorithm for each model. Accordingly, the 100 features from individual model are considered. Finally, each feature is merged to form a strong feature set. The selection of subcategory of optimal features are nominated from provided dataset. In the case of machine learning models, these tasks are important for image classification CB algorithm. The overall accuracy of the learning model shot up with the help of optimized features. Initially, the classifier was processed freely and 9 major features from each algorithm were calculated. Among 4 subset features, only 6 features are selected which is the most common [67, 68]. By this way, the hyper parameter tuning is performed. Using CB the hyper parameters are tested. The learning algorithms provide greater performance of evaluation metrics.

### 3.6 VC using CatBoost algorithm

In the final step, the selected vehicle features are used to classify the vehicle based on classes and model. The number of classifiers including SVM, NB, RF and CB are then used for training and their performance is evaluated on test data. Later on, the data is tested on classifier models. In this paper, CB algorithm is implemented for VC with inter class and intra class variability in the complex environment. CB is a supervised ML technique used by train tooled and used for classification and regression. As its name suggests, it works with categorical data and is used for boosting. Gradient boosting processes where the decision trees ordered iteratively. This technique has an advantage of consuming both categorical and non-categorical variable without pre-processing. Initially, the dataset of vehicle images is denoted by A, calculated in Eq (3)


$$A={\left\{\left(b_{d},c_{d}\right)\right\}}_{d=1,2,\ldots n}$$ (3)

The random vector of the vehicle feature selection is given in (4)

$$b_{d}=\{b\begin{array}{c} 1\\ d \end{array}\ldots ..b\begin{array}{c} e\\ d \end{array}\}$$ (4)

The target vehicle variable c_d_ ϵ f may be in the form binary or numerical,

b_d_, c_d_ are variable which contain unconventional and similar attribute accumulate to unspecified m(.,.) distribution G: H^e^ → H which contain minimum loss function. The loss function is shown as (5)

λ(G)=JK(C,G(b)) (5)

K() → smooth loss function.(b, c) are test example with m independently training set. Alternatively, the sequence approximation is given in (6)

$$G_{n}:H^{i}\to H\;are\;gradient\;boosting\;approximation(n=0,1\ldots ..)$$ (6)

G^n-1^ → obtained from previous application, the formula is given in (7)

$$G^{n}=G^{n‐1}+\alpha \;O^{n},\;\alpha \to step\;size$$ (7)

O^n^: H^i^ → H (fundamental predictor)

Taken from the group of function O in order to minimize the expected loss. It is expressed in (8)

$$O^{n}=argmin\;k(G^{n‐1}+O)=argminJK(b,\;G^{n‐1}(b)+o(b))$$ (8)

o → O minimization problem. The false gradient step is given by (9)

$$k(g^{n‐1}+o^{n})\;at\;g^{n‐1}\;as\;a\;negative\;gradient\;step$$ (9)

Gradient step G^n^ → it will be chosen as g^n^(a) approximate p^n^(a, b) and it is denoted in (10)

$$p^{n}(a,\;b)=\frac{\delta k(b,q)}{\delta g}|q=g^{n‐1}(a)$$ (10)

Least squares approximation is used in (11)

$$O^{n}=argmin\;J{\left(‐p^{n}(a,\;b)–O(a)\right)}^{2}$$ (11)

Decision tree

H^e^ → several disjoint nodes

The values of some splitting attributes denote as “r”

Attributes are of binary variable used to identify the same feature b^d^ exceeded

Some threshold S, that is,

r = T when the b^d^ > n

b^d^ are numerical or binary features having n = 0.5^2^

In the regression task, the each terminal region are represented as a leaf on the tree and assigned a value that estimates the response variable C. This approach effectively addresses classification challenges through decision trees. Hence, the decision trees O is represented in (12),

$$O(a)=\sum_{u=1}^{U}V_{u}T\{b^{d}\;\epsilon \;H_{u}\}$$ (12)

H_u_ is disjoint region about to leave the tress with effective and efficient way to deal that tree V is to subcategory $b_{d}^{i}$ of the d^th^ are the sample training dataset having one feature is associated with target spaces. The features are in the form of numbers. An intermediate experiment target y conditioned by category ${\hat{b}}_{d}^{v}$. Commonly, it estimates experiment target y conditioned by category and it given in (13). The vehicle classification task is formulated in (14)

$${\hat{b}}_{d}^{v}\approx J(C/b^{v}=b_{d}^{v}$$ (13)

$$W(c=1/b^{v}=R)=0.5\to classification\;task$$ (14)

Training vehicle dataset is given in (15)

$${\hat{b}}_{d}^{v}=\frac{c_{d}+rw}{1+r}$$ (15)

One split based on threshold is shown in (16)

$$n=\frac{0.5+rw}{1+r}$$ (16)

Which perfectly classify all training equation

For testing, the value of greedy target statistics is W. The predicted model are assigned as zero for C<n and 1 for the accuracy having 0.5 in 0 and 1 cases. The desired property for target statistics are formulated, it represented in (17)

$$W/J({\hat{b}}^{v}/c=x)=J({\hat{b}}_{d}^{i}/c_{d}=x)$$ (17)

b_d_, c_d_ are the d^th^ training sample

J(${\hat{b}}_{d}^{v}$/c_d_) = $\frac{c_{d}+rw}{1+r}$ and J(${\hat{b}}_{d}^{v}$) = W are different. There are many way to restrict the condition shift. To calculate the target statistics for b_d_ on the A_d_⸦ A\{b_d_} which exclude the b_d_:

$${\hat{b}}_{d}^{v}=\frac{\sum_{b_{u}}\in E_{d}}{\sum_{b_{u}}\in E_{d}}\frac{T\{b_{u}^{v}=b_{d}^{v}\}.C_{d}+rw}{T\{b_{u}^{v}=b_{d}^{v}\}+r}$$ (18)

In Eq (18), ${\hat{b}}_{d}^{v}$ is considered as χ. Based on target statistics of the features, the vehicles are classified.

## 4. Experimental results and discussion

**Fig 6:**
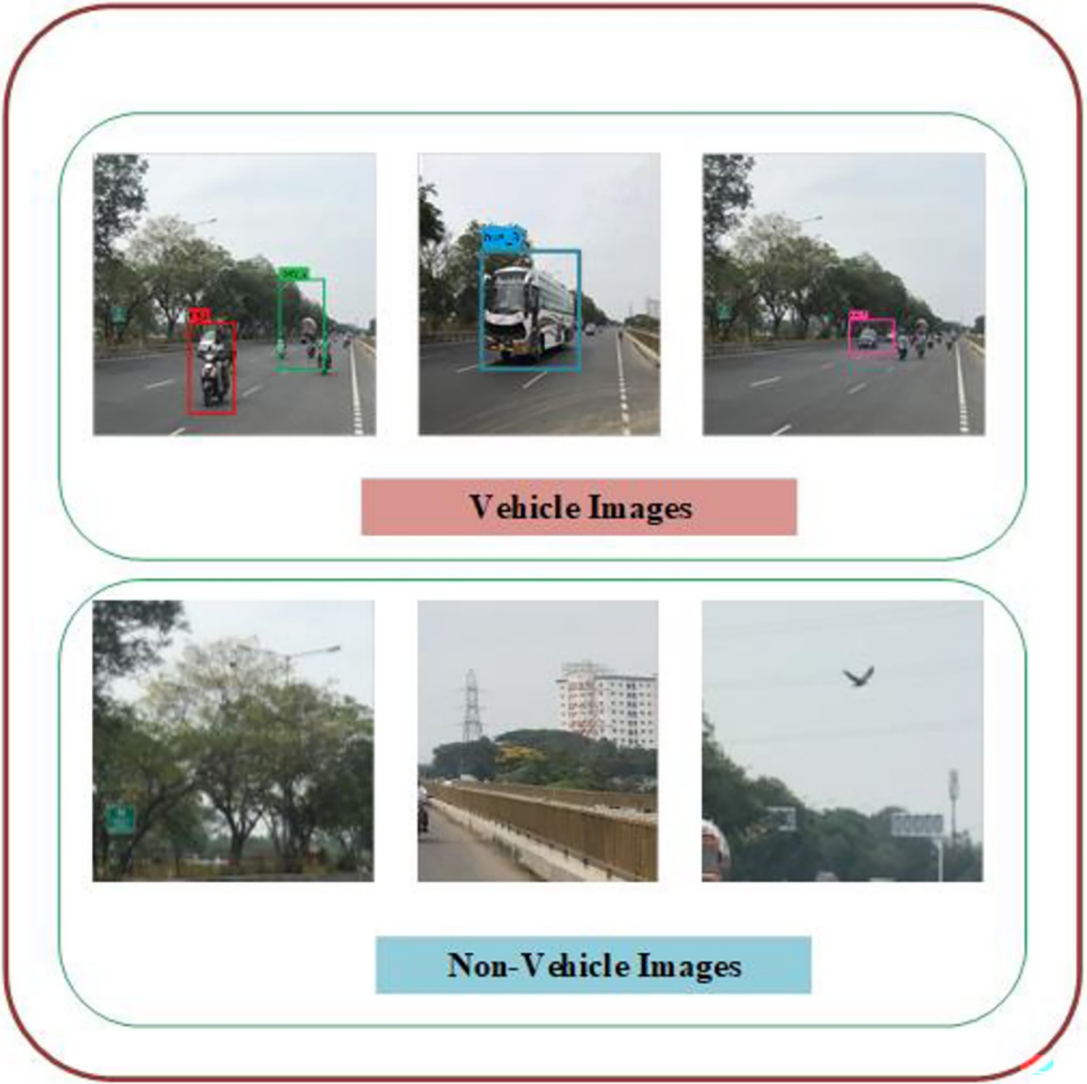
Detection of vehicle with bounding box and class label output.

**Fig 7:**
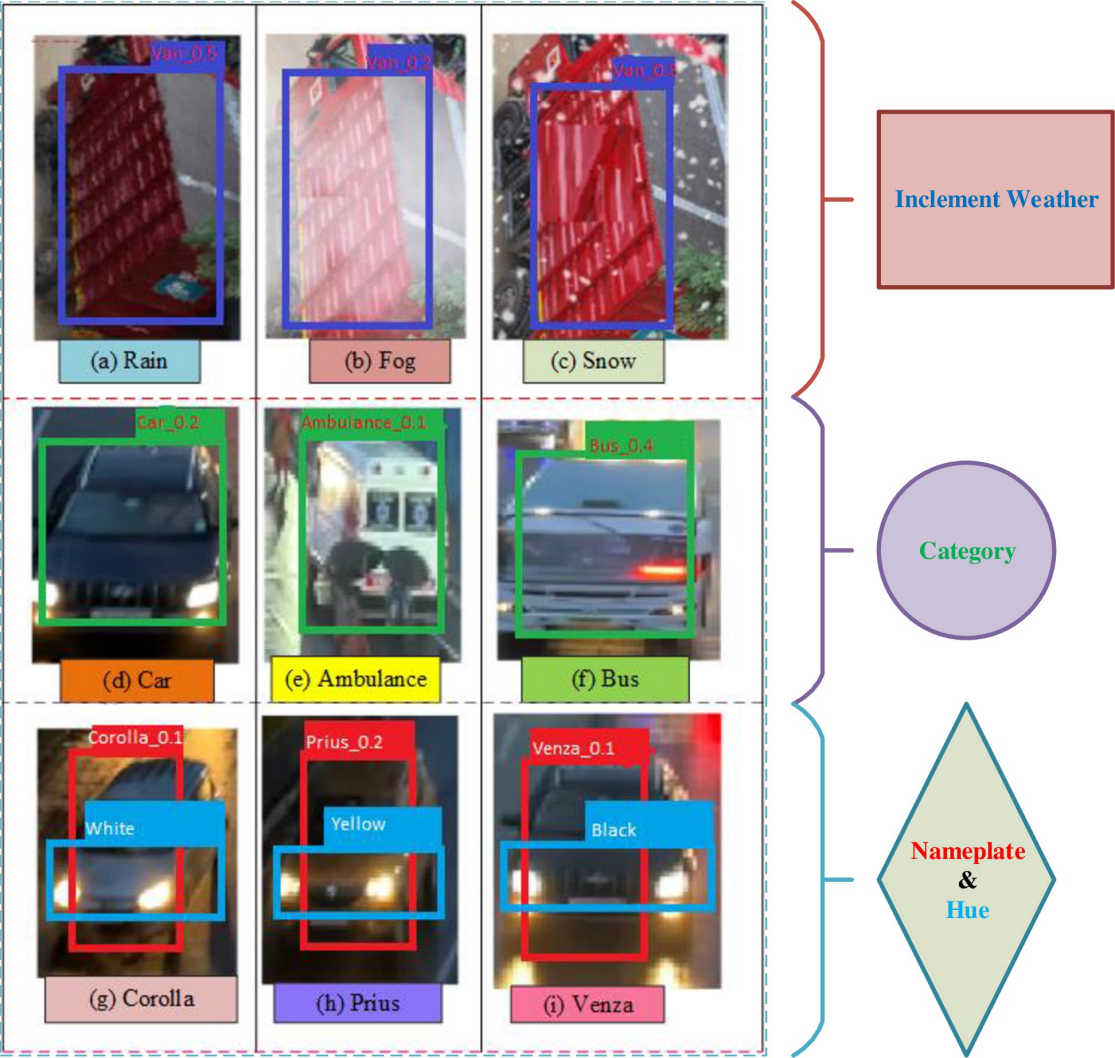
Result of VC under different lighting conditions.

This section presents the implementation results and analysis of the proposed approach. VGG16 DL model is implemented, which consists of 16 layers of convolution and fully connected layer with 224*224 input pixel images containing 3*3 filter sizes. The probability of classes to output layer is performed with the activation function. For our proposed VC system, 80% and 20% of data is used for training and testing purposes. In sequence manner, the training and testing are equally divided. From the given vehicle dataset, the input images and extracted images are shown in Fig 6. Fig 7 shows the output result of VC under inclement weather, types, color and model of the vehicles. In Table 2, the comparison between the proposed approach and existing methods is conducted across different vehicle dataset with various classifiers.

**Table 2:** Shows the comparison of various vehicle dataset with different classifier model.

Dataset	Classifier	Precision	Recall	F1-score	Sensitivity	Specificity	Accuracy
Stanford car dataset	SVM	0.86	0.88	0.86	85.24	0.65	89.42
	KNN	0.71	0.75	0.72	84.24	0.75	92.46
	RF	0.81	0.83	0.81	78.69	0.83	87.35
	**CB**	**0.92**	**0.91**	**0.91**	**91.58**	**0.96**	**98.67**
Vehicle–Rear dataset	SVM	0.76	0.78	0.76	81.56	0.68	68.62
	KNN	0.65	0.68	0.66	74.78	0.85	85.38
	RF	0.84	0.85	0.84	79.68	0.92	94.29
	**CB**	**0.93**	**0.92**	**0.92**	**93.25**	**0.94**	**95.35**
MVVTR dataset	SVM	0.8	0.81	0.8	89.55	0.86	93.68
	KNN	0.76	0.74	0.74	78.86	0.71	89.43
	RF	0.78	0.79	0.78	84.86	0.81	85.85
	**CB**	**0.91**	**0.9**	**0.9**	**92.34**	**0.93**	**95.84**
Indian Vehicle dataset	SVM	0.93	0.89	0.9	86.63	0.8	81.14
	KNN	0.89	0.78	0.83	79.25	0.76	74.35
	RF	0.85	0.84	0.84	82.61	0.78	79.26
	**CB**	**0.94**	**0.93**	**0.93**	**93.34**	**0.94**	**93.58**
TRANCOS dataset	SVM	0.85	0.86	0.85	70.45	0.76	68.44
	KNN	0.76	0.79	0.77	85.79	0.65	85.93
	RF	0.81	0.82	0.81	91.68	0.84	94.06
	**CB**	**0.92**	**0.93**	0.92	**92.57**	**0.92**	**95.45**
Thai Vehicle Classification Dataset	SVM	0.68	0.7	0.68	85.83	0.83	81.58
	KNN	0.85	0.85	0.85	72.39	0.72	74.35
	RF	0.92	0.91	0.91	82.46	0.78	79.37
	**CB**	**0.95**	**0.95**	**0.95**	**91.84**	**0.87**	**89.93**
2023 Car Model dataset	SVM	0.81	0.83	0.81	68.38	0.86	78.86
	KNN	0.74	0.72	0.72	85.75	0.71	84.28
	RF	0.79	0.78	0.78	92.69	0.81	89.22
	**CB**	**0.96**	**0.95**	**0.95**	**93.56**	**0.92**	**94.59**
**UFPR-ALPR dataset**	SVM	0.65	0.68	0.66	85.63	0.88	68.37
	KNN	0.84	0.85	0.84	76.83	0.75	85.59
	RF	0.93	0.94	0.93	81.56	0.83	92.25
	**CB**	**0.98**	**0.97**	**0.97**	**98.87**	**0.98**	**98.89**
VehicleX dataset	SVM	0.65	0.67	0.65	65.36	0.83	68.43
	KNN	0.84	0.85	0.84	84.45	0.72	85.52
	RF	0.93	0.92	0.92	91.73	0.78	92.81
	**CB**	**0.93**	**0.94**	**0.93**	**92.36**	**0.92**	**95.36**
CompCars dataset	SVM	0.86	0.85	0.85	83.45	0.86	86.25
	KNN	0.71	0.72	0.71	72.86	0.79	79.68
	RF	0.81	0.82	0.81	78.86	0.82	82.42
	**CB**	**0.91**	**0.97**	**0.93**	**93.21**	**0.91**	**96.35**

Fig 7(A)–7(C) shows results of the VC under adverse conditions. Fig 7(D)–7(F) show the result of different vehicle types. Fig 7(G)–7(I) shows the result of various color and model of the vehicles. The sample images are displayed with bounding box and label.

**Fig 8:**
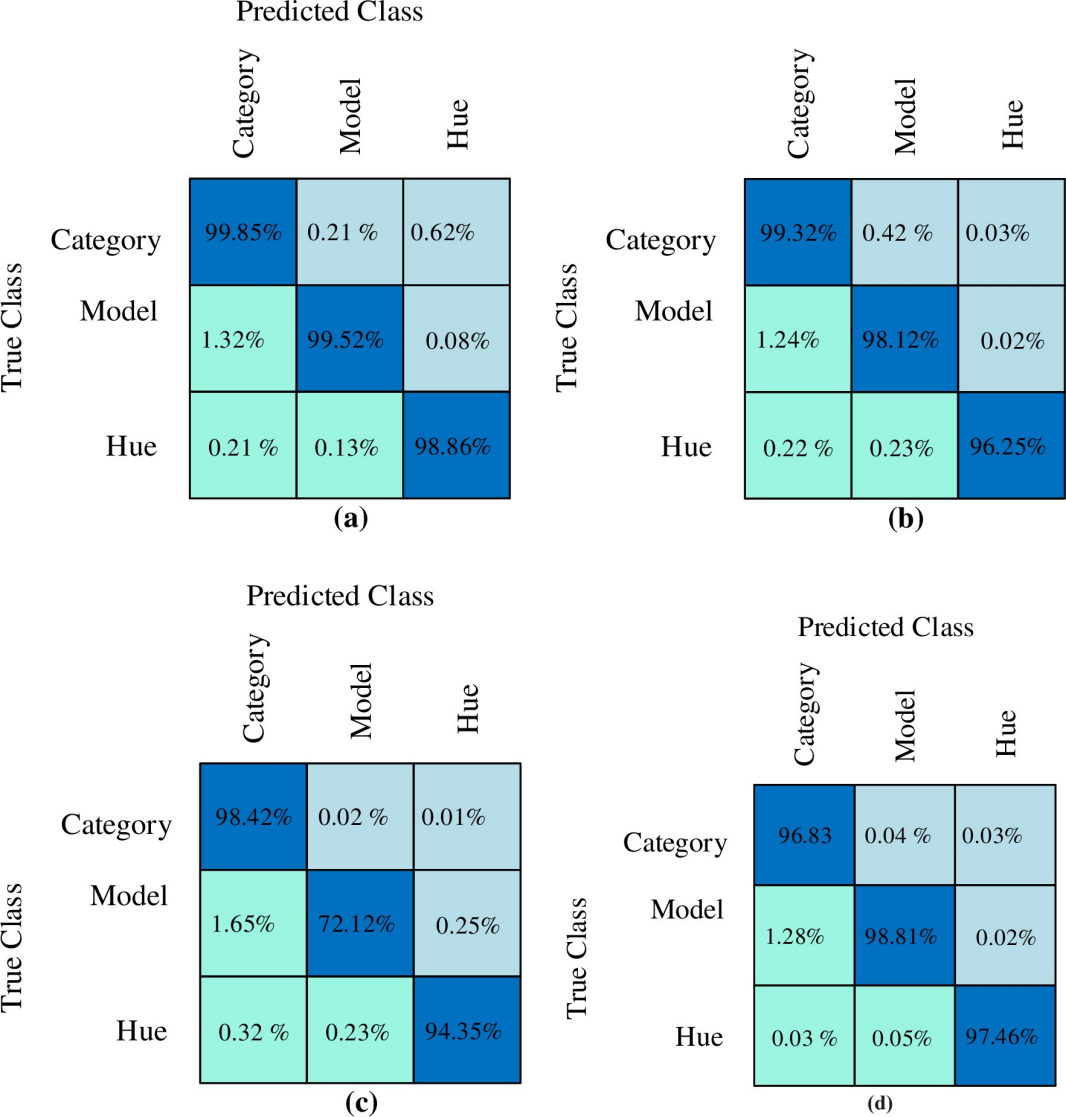
Confusion matrices from UFPR-ALPR dataset showing the relative performance. (A) CB (B) RF (C) KNN (D) SVM.

**Fig 9:**
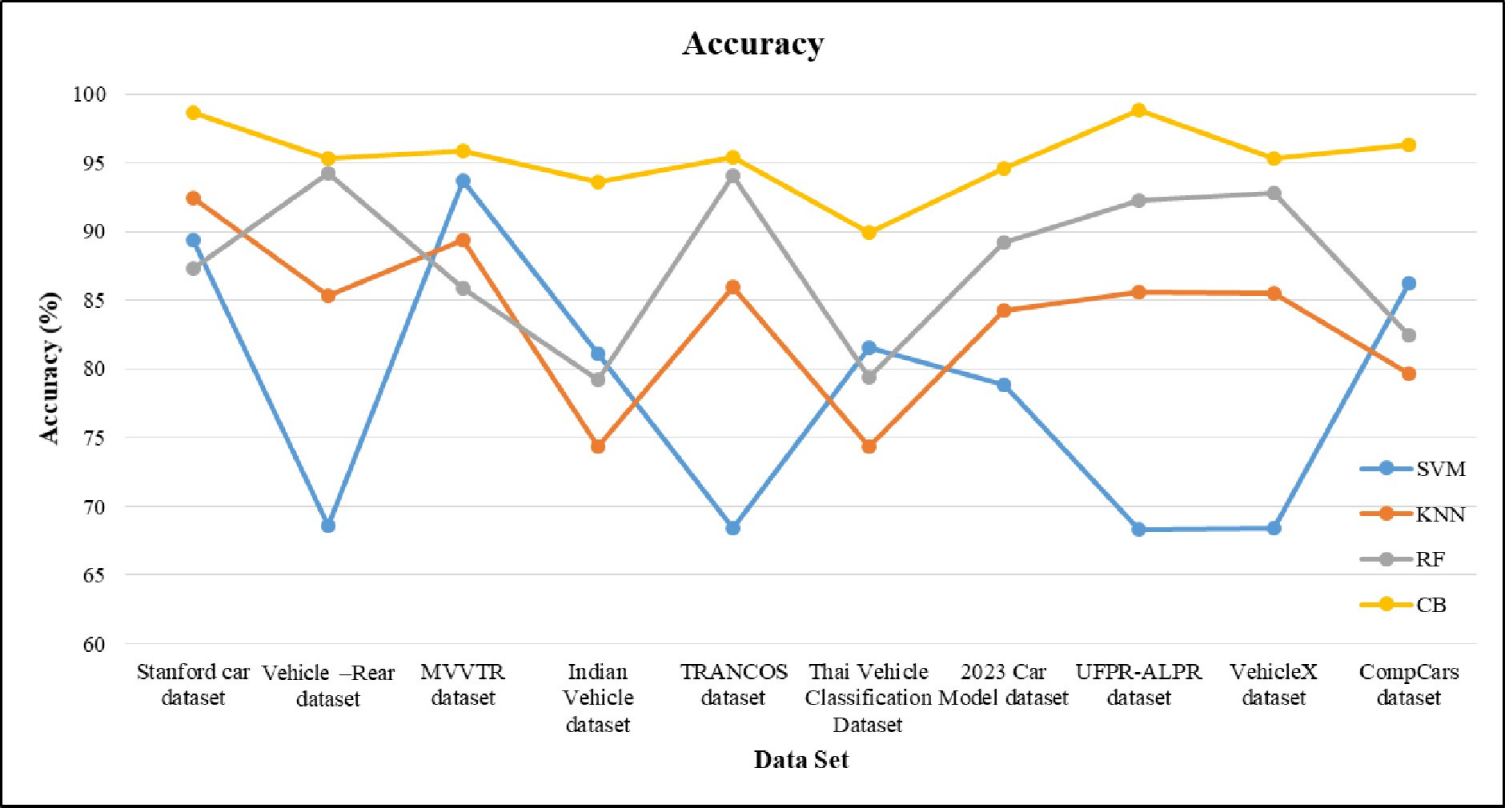
Accuracy comparison of proposed model over different dataset for VC.

To define the classification performance of the classifier, the confusion matrix is implemented. In Fig 8, confusion matrices of each classifier performance on the UFPR-ALPR dataset. From the figure, the confusion matrices for CB and RF is more or less identical. However, CB yield the best performance for VC task which is less prone to overfitting. Similarly, the accuracy for VC are also compared with different classifier namely SVM, KNN, RF and CB in Fig 9. RF and KNN attain the maximum accuracy level of classification greater than 85% when compared to KNN. The SVM gain accuracy value less than 70%. Our proposed method achieves the optimal accuracy result 98.89% using CB algorithm over UFPR-ALPR dataset.

**Fig 10:**
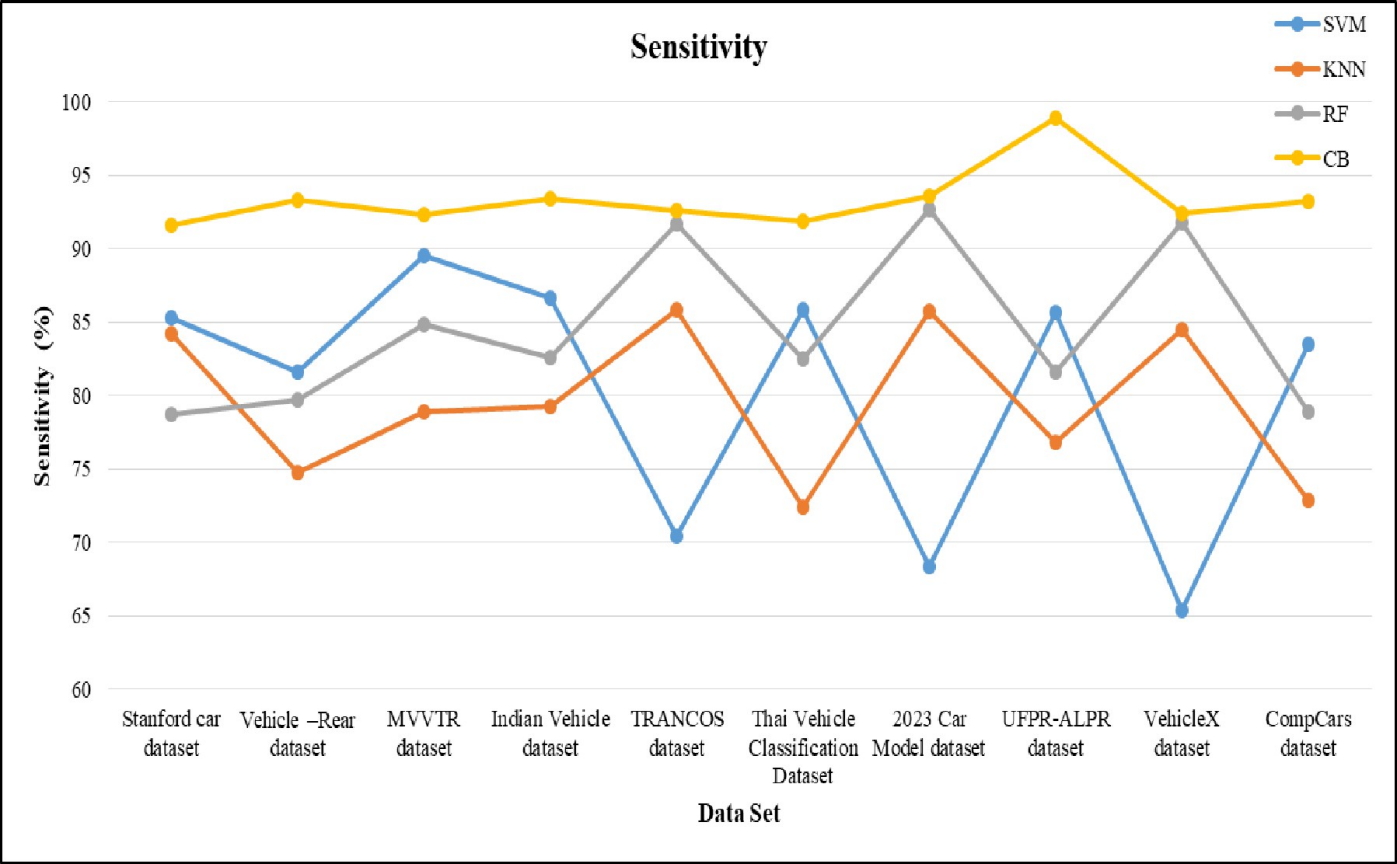
Sensitivity of proposed model over different dataset for VC.

**Fig 11:**
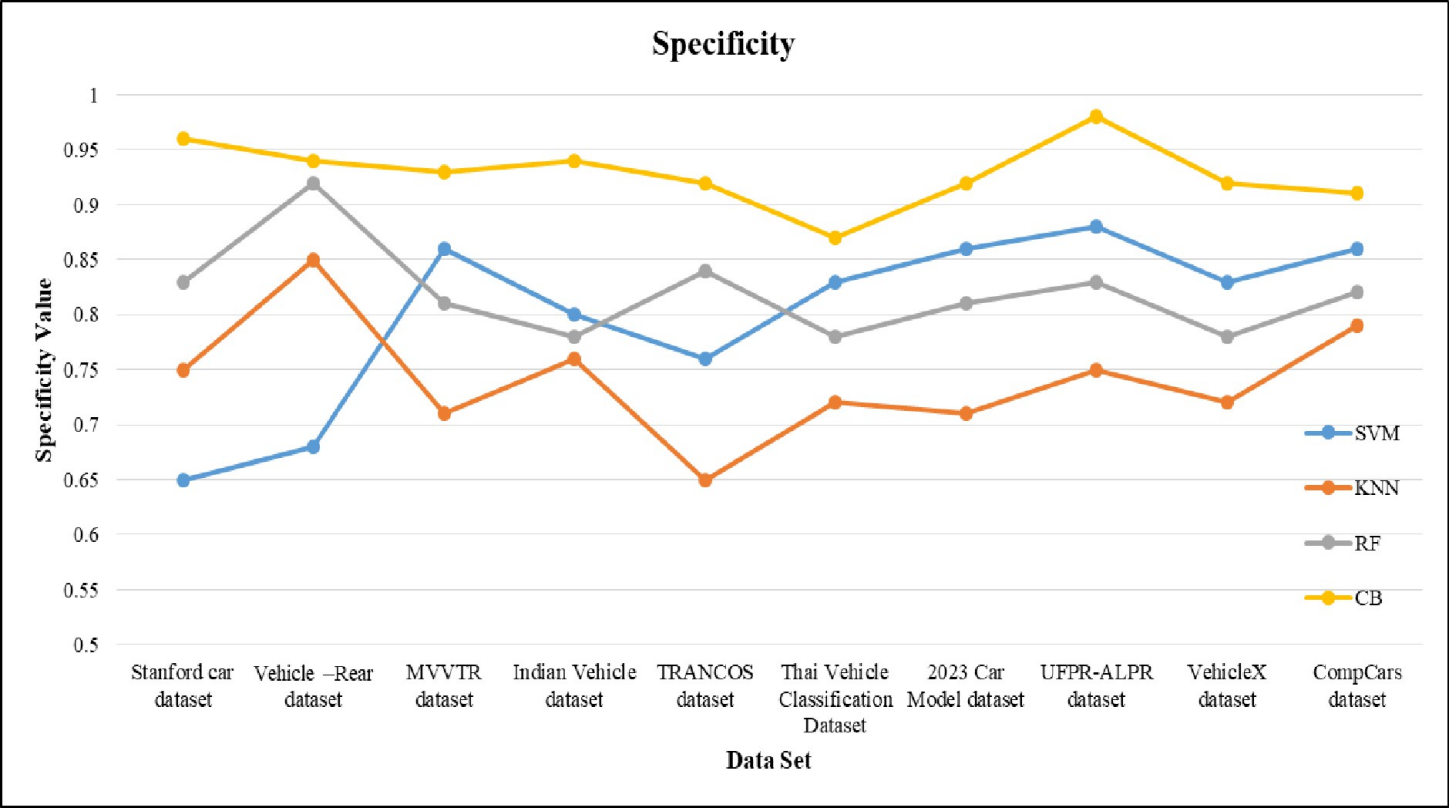
Specificity of proposed model over different dataset for VC.

The sensitivity of the proposed machine learning model is shown in Fig 10. For the proposed method, the sensitivity gained by 98 and for SVM, KNN and RF the values are nearly 86, 77 and 92. From the result of sensitivity obtained, KNN and RF have less sensitivity when compared to proposed approach. The specificity is compared in Fig 11. The specificity values are 0.75, 0.83, 0.86 and 0.98 for KNN, RF, SVM and CB respectively. The CB gains higher specificity values when compared to rest of the given classifier.

**Fig 12:**
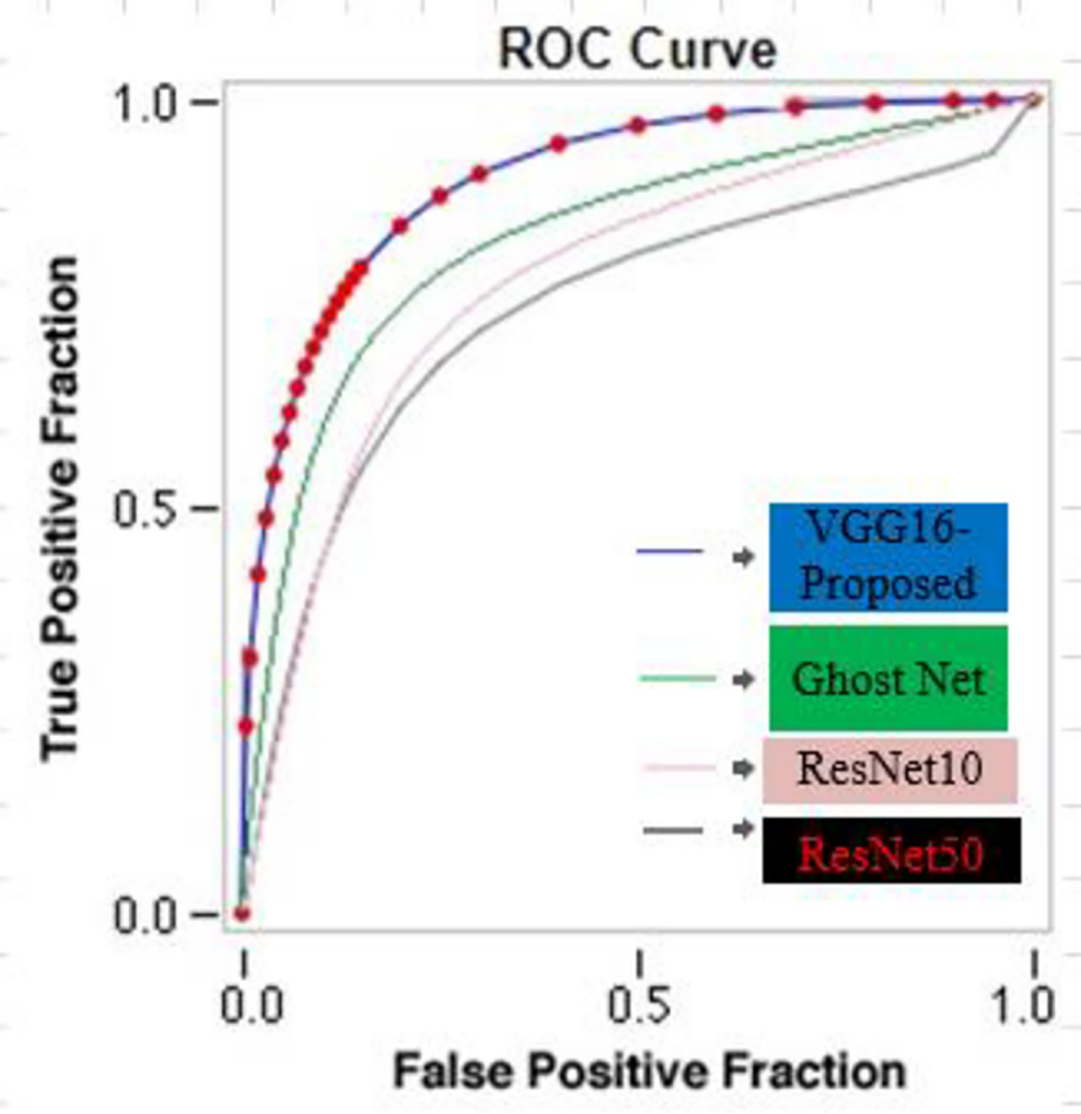
ROC curves of the test results of the different networks.

The area under curve for VGG16 approach is shown in Fig 12. For the proposed method, the area under curve value obtained is 99.56 and for traditional approaches such as ResNeXt50, ResNet10 and GhostNet gain the value of 70.23, 71.78, 84.65 and 88.01. Hence the proposed approach has a better result when compared to the existing methods. ResNeXt50 gain the area under curve less than 80 whereas ResNet10 and GhostNet gain the area under curve is more than 80.

**Fig 13:**
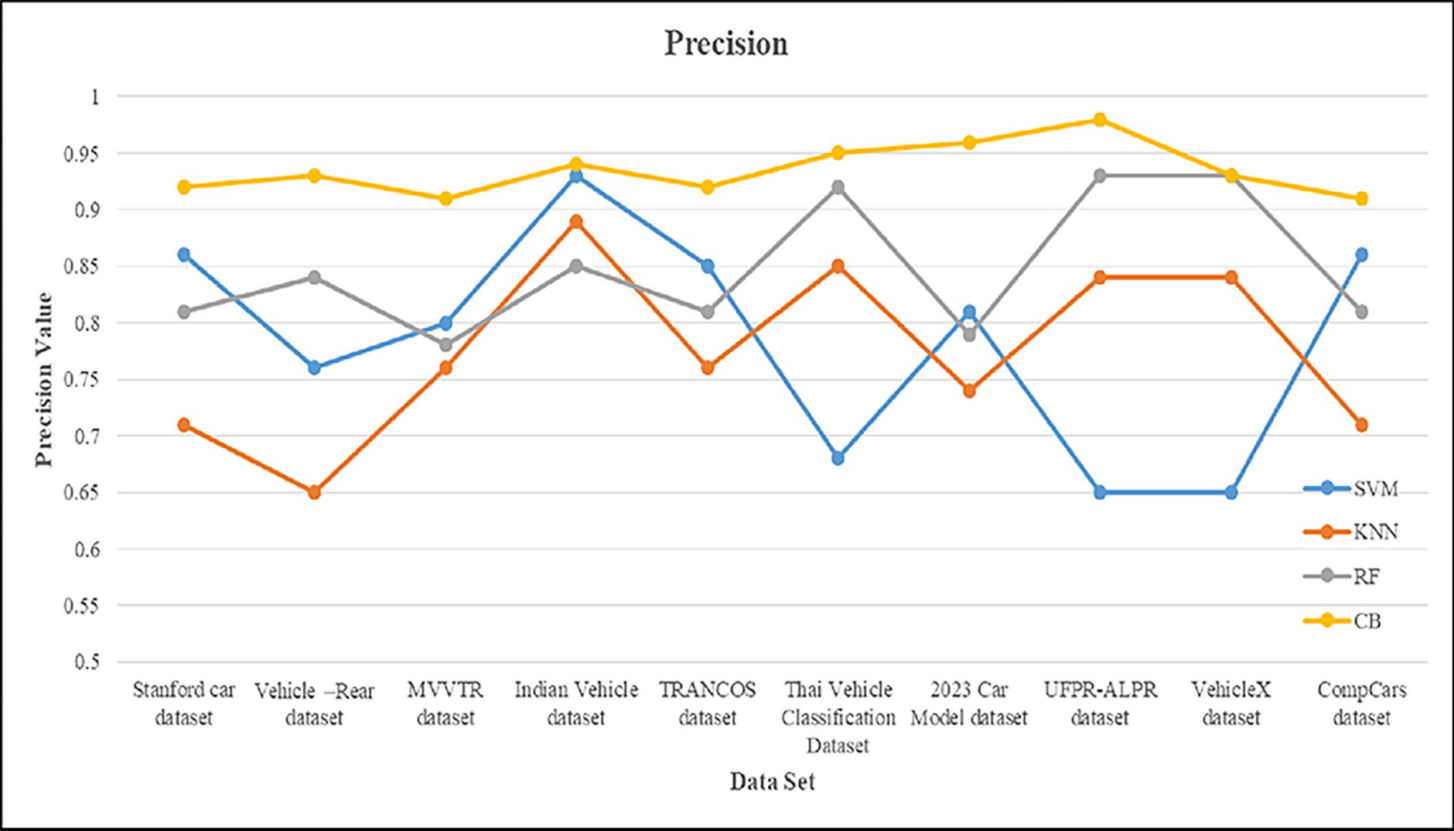
Precision comparison of various model over UFPR-ALPR dataset.

The precision for proposed approach is compared along with the existing methods such as SqueezeNet, ResNeXt50, ResNet10 and VGG16 is shown in Fig 13. The proposed algorithm gain 99.98 of precision value. The SqueezeNet and ResNeXt50 are 70 ranges whereas ResNet10 and GhostNet are 80 ranges. VGG16 gain higher value of precision around 88.7 when compared to the other existing approaches.

**Fig 14:**
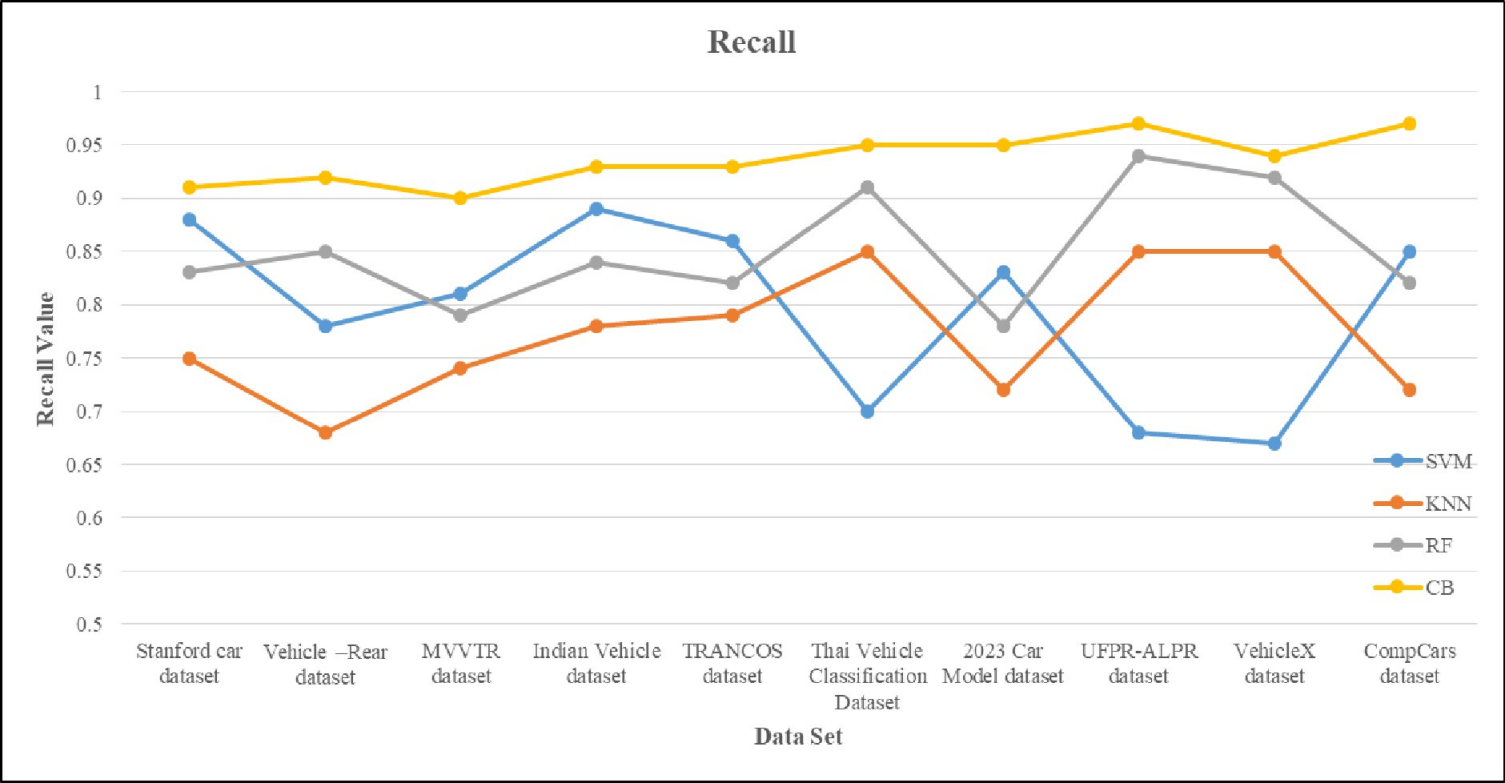
The recall graph of the complex dataset of VC using VGG16 with different classifier.

**Fig 15:**
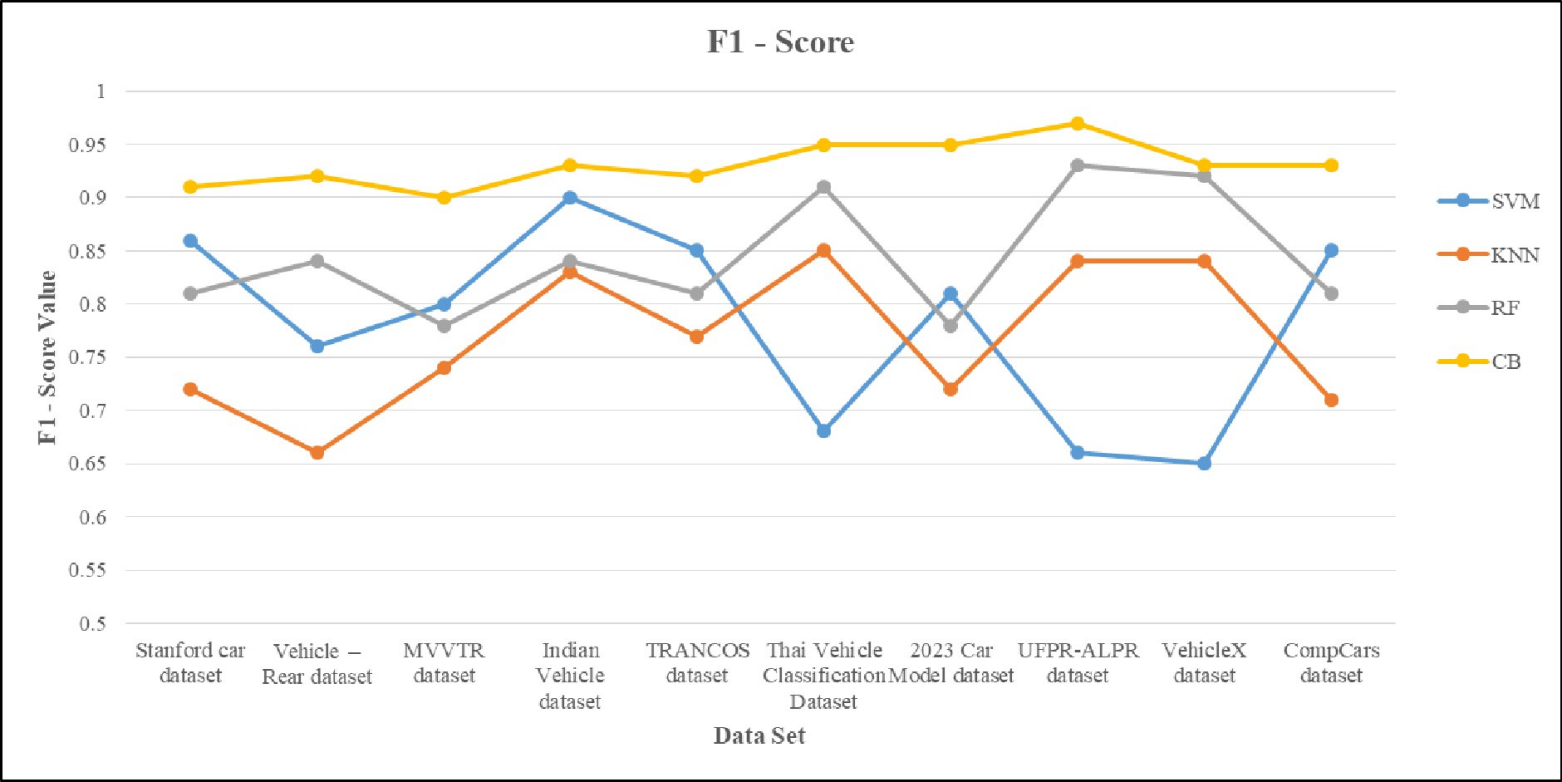
The f1-score graph is highlighted on various dataset for VC using ensemble classifier.

The accuracy is compared with the performance of different networks such as SqueezeNet, ResNeXt50, ResNet10 and GhostNet containing error rate. The error is applied to test the performance of the proposed model. If the error rate is high, then the performance of the model is weak. By boosting error upto 2, there would be demote in accuracy performance. The existing networks such as SqueezeNet and ResNet10 have less performance due to inclusion of error whereas reaming network gain the performance of 80%. The performance of the proposed technique is unaffected based on error input. Figs 14 and 15 show the recall and f1 score of the proposed methods over different classifiers. In this SVM and KNN gain less recall when compared to RF. The RF obtained the recall of 91.23% and proposed is about 93%. The F1 score is high in case of SVM of about 95% but in compared to SVM, the proposed namely CB gain of 98.89%.

The proposed VC uses CB algorithm which provides classification of vehicle based on color, inclement weather, types and model. If more parameters are included then the model is needed for tested with specified approach. The feature extraction with the equivalent representations and handling input variable size are more accurate. Since for the classification of the images the input layer must be in fixed size. Finally, classification is invoked with CB which supports the categorical feature. This led to lower the information loss and avoids overfitting data.

## 5. Limitation

Using ensemble CB model for complex dataset helps governing the complex images and text data. But the stumbling block is that these methods need more utilization power because of various stages of process. This might delay the execution process with large scale dataset. Also, there is an intricate of the model procuring more attentive on minimum particulars in the training data. This led to languishing while get grips with the various data when there is unavailability of training data.

## 6. Challenges and future scope

Though there are dominant mechanisms for vehicle classification using CNN, there are some bottleneck in employing these methods. The major hindrance to overcome vehicle classification is the necessity of intensive training in neural networks methods. An accurate training is mandatory for the processor to perform more precise investigation and interpretation. This led to endless time. To overcome these issues the hardware must be robust and potent. The robust hardware and GUI are the most prerequisite for vehicle detection and classification using deep models. Another obstacle is having more processing time and power. The variance in vehicle dimension in complex environment is common provocation to be faced. Image pixel calculation less than millimeter size is generally difficult to analyses and lead to error in early stage of detection. It can be very tedious to differentiate shape, color, texture and in many cases. It is highly challenging to categorize and analyze the vehicle image due to interclass variation. Some datasets may be uneven. Many vehicle images are found but some are uncommon ones. As a result, reducing the vehicle images from visual features are great challenging. Image segmentation under crucial environment is the greatest task. Therefore, peculiar algorithms are used for segmentation processes. Nowadays, the emerging research on vehicle detection and classification is more pivotal. The current research is mostly focused on specific problems of image classification. To address traffic police and toll collection concerns, future research on vehicle classification may be highly focused on amalgamating entire images of vehicle. Fetching uninterrupted shots will accelerate the picture acquiring process. The concept of preprogrammed mechanized is one that was recently organized. This highlights unsupervised learning to recognize the features and inquire into comparison between individual images and dataset. There are more research and studies are carried out on deep model.

Future research could focus on numerous approaches to enrich the classification of vehicle images by advanced CNN with optimization algorithm. Expanding the complex vehicle dataset, fusing additional modal like local transportation data, decision making interpretability model development, analyzing transfer learning and particular domain versatility, incorporating virtual and real learning processes into application, amplifying the algorithm for real time application on wireless handset, research work on validation processes. Using transfer learning processes, pre trained model are rearranged to specific parameter of the dataset. Depending on the application, the model is trained on compact dataset. These methods are used to learn mobility visualization features from source to destination. This will further reduce the quantity of features of the data in major areas. A few shot learning is useful for learning small features. Whereas greater quantity of small features of data processing makes the system costlier due sampling less classes. For tackling the real time classification of images and learning labeled data issues, combination of few shots and transfer learning are applicable. The major objective of these future work is to optimize the efficiency of the model, applicability and less power consumption of detecting and classifying vehicle under various factors.

## 7. Conclusion

In this paper, CB algorithm is proposed for VC from the different complex dataset. Initially, the vehicle images are pre-processed with contrast enhancement technique. The external and internal features are segmented using Mask R-CNN and VGG16DL is used for the extracting the nominal features. From the segmented vehicle images, the most prominent features are extracted. Autoencoder is used for selecting features which result in compressed data. Finally, CB is implemented for classifying the vehicle based on types, color, and model under adverse weather. The experiment is carried out over different large scale vehicle datasets over various classifiers. The proposed performance is analyzed with performance metrics such as accuracy, recall, specificity, precision, sensitivity, and f1-score and compared with existing approaches. The performance of the proposed method is evaluated using metrics such as accuracy, recall, specificity, precision, sensitivity, and F1-score. Furthermore, a comparative analysis is conducted with existing approaches. By using explicit operational relationship of vehicle images, the accuracy for VC is achieved by 98.89%. The experimental results of complex large scale vehicle dataset demonstrate the CB algorithm surpass existing classifiers. For further extension, the experiment can be carried out in hardware implementations.
